# Genetic Prediction of Osteoporosis by Anti-Müllerian Hormone Levels and Reproductive Factors in Women: A Mendelian Randomization Study

**DOI:** 10.1007/s00223-024-01220-5

**Published:** 2024-05-14

**Authors:** Yuan Li, Jinquan Lai, Wenbo Wu, Shuyi Ling, Yuqing Dai, Zhisheng Zhong, Xiaodong Chen, Yuehui Zheng

**Affiliations:** 1 Shenzhen Traditional Chinese Medicine Hospital, Shenzhen, China; 2 The Fourth Clinical Medical College of Guangzhou University of Chinese Medicine, Shenzhen, China; 3 Shenzhen Luohu Hospital of Traditional Chinese Medicine, Shenzhen, China

**Keywords:** Anti-Müllerian hormone, Female reproductive factors, Osteoporosis, Bone mineral density, Mendelian randomization

## Abstract

Previous observational studies have suggested that anti-Müllerian hormone (AMH) and reproductive factors are linked to reduced bone mineral density (BMD) and an increased risk of osteoporosis (OP) in women. However, related studies are limited, and these traditional observational studies may be subject to residual confounders and reverse causation, while also lacking a more comprehensive observation of various reproductive factors. Univariate and multivariate two-sample Mendelian randomization analyses were conducted to determine the causal associations of AMH levels and six reproductive factors with BMD and OP, using the random-effects inverse-variance weighted method. Heterogeneity was assessed using Cochran’s Q-statistic, and sensitivity analyses were performed to identify causal correlations. Age at menarche (AAM) was negatively associated with total body BMD (TB-BMD) in females aged 45–60 and over 60 years, as well as with heel bone mineral density (eBMD). Conversely, age at natural menopause (ANM) was positively associated with TB-BMD in the same age ranges and with eBMD. ANM was only causally associated with self-reported OP and showed no significant correlation with definitively diagnosed OP. Neither AMH level nor other reproductive factors were significantly associated with a genetic predisposition to BMD at any age and OP. Later AAM and earlier ANM are significantly genetically causally associated with decreased BMD but not with OP. AMH levels, length of menstrual cycle, age at first birth, age at last birth, and number of live births, in terms of genetic backgrounds, are not causally related to BMD or OP.

## Introduction

Osteoporosis (OP) is a systemic metabolic bone disease that causes chronic pain. It can even lead to immobility and reduce life expectancy due to its most severe complication, osteoporotic pathological fracture [1]. About 9 million osteoporotic fractures occur annually worldwide, and they cause more disability-adjusted life-years loss than common cancers except lung cancer in Europe [2]. OP has become the fourth major noninfectious pathology following cancers, cardiovascular disorders, and stroke.

Females face a significantly higher risk of OP (40–50%) than males(13–22%) over the age of 50 years [3, 4]. Perimenopausal or postmenopausal estrogen deprivation and consequent structural changes in bone mass or bone tissue are known causes of OP in women. Estrogen has been shown to inhibit osteoblast apoptosis and osteoclast formation and has been proven to be a key regulator of bone metabolism with pleiotropic effects [5]. Anti-Müllerian hormone (AMH), similar to estrogen, is a hormone secreted by ovarian granulosa cells. However, AMH secretion only occurs at earlier developmental stages of follicles, allowing it to serve as an earlier indicator than estrogen for detecting the onset of menopause or the decline of ovarian function [6]. Moreover, the changes in AMH levels throughout a woman's life align with the trends in bone mass changes, meriting further exploration into its potential direct effects on female bone metabolism. Although Karlamangla et al. found that lower AMH levels were associated with reduced bone mineral density (BMD) in premenopausal and postmenopausal women, this study did not correct for the confounding effects of estrogen and could not demonstrate the causal relationship therein [7]. Therefore, a more in-depth study is warranted to clarify the relationship between AMH levels and BMD or OP.

In addition to menopause, other reproductive factors such as the menstrual cycle and conception, can also affect the endocrine system and potentially affect bone metabolism. Mounting evidence demonstrated that female bone metabolism is significantly influenced by later menarche, a shorter time from menarche to menopause, and a higher number of births [8–10]. Yet, despite adjustments for confounders such as weight and race, these traditional observational studies remained sensitive to confounding bias, and the interconnections and effects of multiple exposures were not considered. Since a comprehensive and reliable observational study would be costly, there is an urgent need to delineate the causal relationship between female reproductive factors and OP using new methods.

To address these concerns, we conducted a Mendelian randomization (MR) study to circumvent the limitations of conventional investigations. Genome-wide association studies (GWAS) have identified a multitude of genetic variants associated with various diseases. MR studies use genetic variations in single nucleotides, named single nucleotide polymorphisms (SNPs), as instrumental variables (IVs) to explore the direct genetic causal relationships between specific exposures and health outcomes. Different genotypes determine different intermediate phenotypes, allowing the association between genotype and health outcomes to be substituted for the effects of exposure factors on outcome indicators. Additionally, this method is based on the Mendelian principle of random assortment, in which parental alleles are randomly distributed to the offspring during meiosis for gamete formation. This ensures that gene-health outcome links are not confounded by postnatal environmental factors, socioeconomic status, or lifestyle habits, while also reducing the interference of reverse causation [11].

Previously, a study investigated the genetic causality between AAM and OP using the univariate Mendelian randomization (UVMR) method, demonstrating that AAM in females may play a causal role in OP etiology [12]. However, it overlooked the potential confounding effects of other female reproductive variables. Multivariate Mendelian randomization (MVMR)) allows for the inclusion of genetic variation for each risk factor in the same model and simultaneous assessment of multiple exposures of interest, minimizing the effect of confounding factors [13]. Therefore, we used both the UVMR and MVMR methods to assess the causal association between several exposures and OP.

## Methods and Materials

### Study Design

Europe has the highest incidence of osteoporotic fractures (34.8%) in the world [2], and hence our analysis was conducted primarily on European women to eliminate the bias caused by population stratification. Female blood AMH levels and six reproductive factors were selected as the exposure factors in this study. The reproductive factors included: age at menarche (AAM), age at natural menopause (ANM), length of menstrual cycle (LMC) (which refers to the interval between two periods), age at first birth (AFB), age at last birth (ALB), and number of live births (NLB). BMD is a clinical criterion for assessing OP and fracture risk [14]. Given that bone loss is closely related to age, both age-stratified BMD and OP (with or without fractures) were determined as outcomes. SNPs that serve as Valid IVs must satisfy the following three key assumptions [15]: First is the relevance assumption: the genetic variants are associated with the risk factor of interest (exposure factors).To meet this assumption, we included only SNPs with genome-wide significance (*p*-value < 5E-8) in the exposures (the threshold may be appropriately lowered when there are particularly few significant sites).To prevent weak instrumental bias, the F-statistic was used to determine the strength of the Ivs and SNPs with an F-statistic < 10 were excluded. The *F*-value was calculated as follows:

$$F = R^{2} \times \frac{N - 2}{{1 - R^{2} }},\, R^{2} = 2 \times \left( {1 - MAF} \right) \times MAF \times \frac{\beta }{SD},\, SD = SE \times \sqrt N$$

Here, N refers to the sample size of the GWAS, SE is the standard error of β, and MAF is the minor allele frequency. The corresponding values were obtained from the original GWAS data.

Second is the independence assumption: there are no unmeasured confounders of the associations between genetic variants and outcomes. To meet this assumption, we first performed UVMR analysis using the SNPs included above. Then, we eliminated SNPs that were significantly (*p*-value < 5E-8) associated with potential confounders determined by previously published studies or in the Pheno Scanner (V2) database (http://www.phenoscanner.medschl.cam.ac.uk/). In this study, SNPs that were highly correlated across various exposure factors were not excluded. The bias resulting from this overlap was corrected in subsequent MVMR analyses.

The last is the exclusion restriction assumption: the genetic variants affect the outcome only through their effect on the risk factor of interest. To meet this assumption, SNPs that were also highly correlated (*p*-value < 5E-8) with the outcomes were removed.

**Fig. 1:**
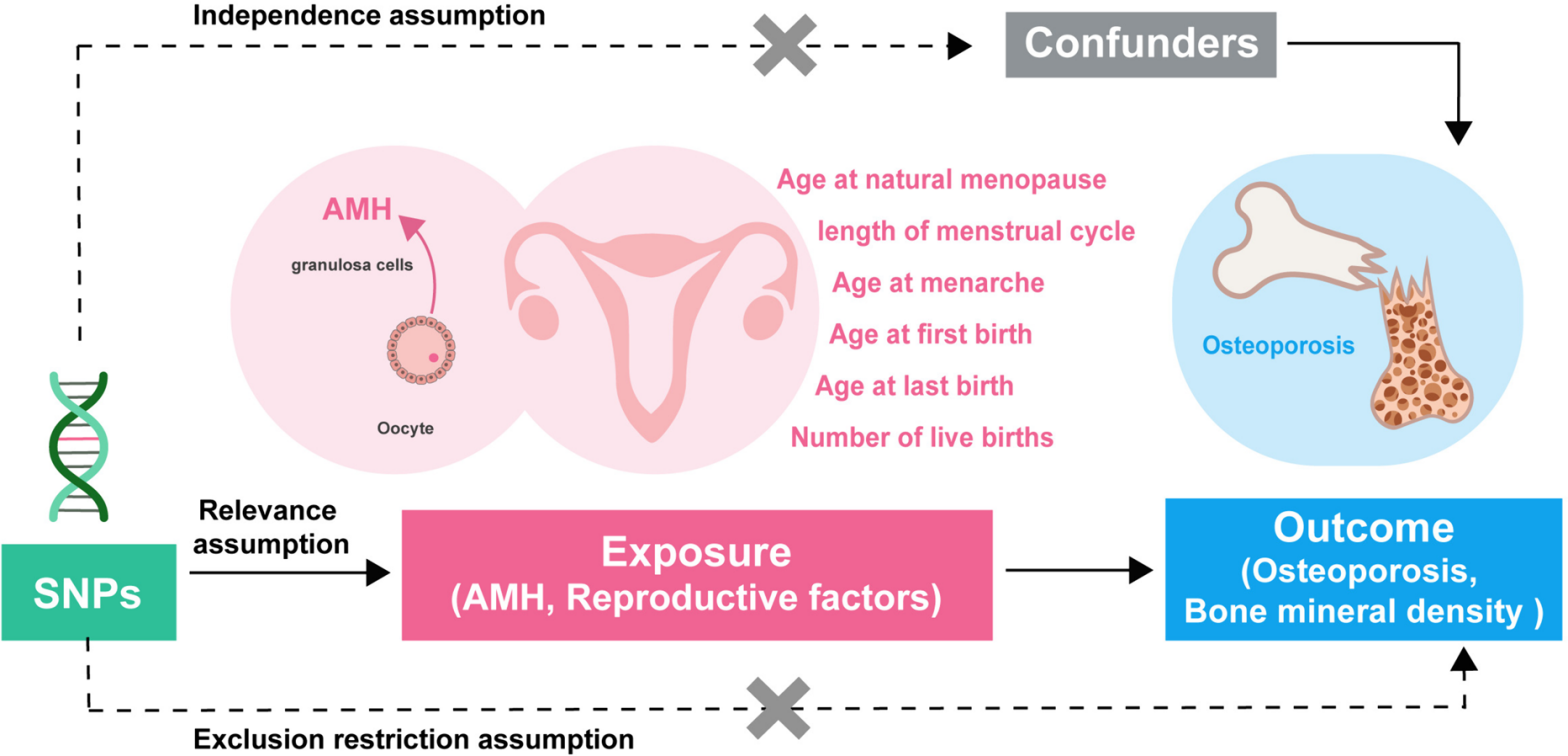
Description of the study design in this Mendelian randomization study and three key assumptions of valid instrumental variables. *SNPs* single nucleotide polymorphisms

Publicly available data were utilized in this study. Informed consent and ethical approvals were obtained in the original studies and were thus not required for this study. A diagram of the study design is shown in Fig. 1.

### Instrumental Variables for Exposures (Women's AMH Level and Reproductive Factors)

Data on AMH levels were obtained from the largest Genome-Wide Association Studies (GWAS) meta-analysis. Verdiesen et al. included data from 7,049 women of European descent, using various ELISA assays to measure circulating AMH levels in serum or plasma samples. A meta-analysis of the aggregated statistics was conducted using a standard error-weighted approach to explore the association between AMH levels and the incidence of breast cancer and polycystic ovary syndrome. 4 SNPs strongly associated with AMH levels were identified during this process[16]. In order to clarify whether AMH can serve as a biomarker for ovarian reserve and the association between female reproductive lifespan and AMH levels before menopause, Ruth K.S. et al. conducted a similar study on 3,344 premenopausal women from 5 cohorts. This study aimed to identify SNPs linked with AMH levels and managed to identify 10 SNPs for AMH using a *p*-value threshold of 5E-6, due to the limited significant sites at the genome-wide significance level (*p*-value 5E-8) [17]. In our study, data on the six reproductive factors were obtained from other GWAS meta-analyses [18–20] or IEU Open GWAS projects (https://gwas.mrcieu.ac.uk/), with the preliminary inclusion criteria being based on the standard of genome-wide significance (*p*-value < 5E−8) for the exposure factors.

Following the criteria outlined in the three key assumptions mentioned previously, to avoid the impact of linkage disequilibrium (LD) on the results, the LD correlation coefficient (r^2^) threshold was set to 0.001, and the distance between two SNPs was set to 10000 kb [21]. Additionally, palindromic SNPs with minor allele frequencies above 0.42 were excluded. Finally, the remaining SNPs were determined as IVs for women’s AMH levels and the six reproductive factors. The IVs for AMH derived from two studies were named **AMH(Verdiesen et al.)** and **AMH(Ruth K S et al.)**, respectively. Detailed information on the relevant data are summarized in Table 1.

**Table 1:** Data sources used in the MR analyses for the current study

Phenotype	Samples	Sex	Age	Ancestry	Source
Exposures					
AMH (Verdiesen et al.)	7049	Females	15.3–48	European	PMID:35274129
AMH (Ruth et al.)	3344	Females	40–49	European	PMID:30649302
AAM	182,416	Females	15.8–79.08	European	ReproGen PMID:25231870
LMC	43,125	Females	Reproductive	European	MRC-IEU
ANM	69,360	Females	40–60	European	ReproGen PMID:26414677
AFB	418,758	Females	28.24–79.07	European	PMID:34211149
ALB	170,248	Females		European	MRC-IEU
NLB	250,782	Females		European	MRC-IEU
Outcomes					
TB-BMD (age 30–45)	10,062	Both	30–45	European(86%)	PMID:29304378
TB-BMD (age 45–60)	18,805	Both	45–60	European (86%)	PMID:29304378
TB-BMD (age over 60)	22,504	Both	Over60	European (86%)	PMID:29304378
eBMD	206,496	Females		European	UK Biobank
OP (FinnGen)	212,778 (3203 cases, 209,575 controls)	Both		European	FinnGen (R9)
OP (UKBB)	194,174 (665 cases, 193,509 controls)	Females		European	UK Biobank
OP with fracture (UKBB)	194,174 (128 cases, 194,046 controls)	Females		European	UK Biobank
OP without fracture (UKBB)	194,174 (630 cases, 193,544 controls)	Females		European	UK Biobank
OP (self-reported) (UKBB)	194,153 (5037 cases, 189,116 controls)	Females		European	UK Biobank

*AMH* anti-Müllerian hormone, *AAM* age at menarche, *LMC* length of the menstrual cycle (the interval between two periods), *ANM* age at natural menopause, *AFB* age at first birth, *ALB* age at last birth, *NLB* number of live births, *TB-BMD* total body bone mineral density, *eBMD* heel bone mineral density, *OP* osteoporosis

### GWAS Summary Statistics for Outcomes (BMD and OP)

Two-sample MR studies require two different genetic datasets to be consistent within a single MR analysis. Our research focused on the relationship between women’s AMH levels and reproductive factors with OP or BMD. Therefore, data for exposure and outcome variables should be sourced from women in two distinct cohorts to prevent sample overlap and to ensure the validity of our findings. Given that our initial inclusion of OP data and individual exposure factors originated from the UK Biobank, we supplemented our analysis with OP data sourced from the FinnGen database (R9) [22].

The femoral neck and lumbar spine are the areas most susceptible to fractures caused by OP, making BMD for these areas the most conventional indicator for diagnosing OP. However, the relevant data we found and certain exposure factors we identified were subject to sample overlap and originated from mixed-sex populations rather than solely derived from females. To minimize sex bias and sample overlap, we included data on heel bone mineral density (eBMD) assessed solely in females and supplemented this with age-stratified data on the total body bone mineral density (TB-BMD) containing information from both males and females. The data for TB-BMD were derived from a meta-analysis that compiled 30 GWAS on TB-BMD to investigate the genetic determinants of TB-BMD variations throughout the lifespan and to test for age-specific effects. This analysis included a total of 66,628 individuals, divided into 5 age groups and we only included data from groups beyond 30 years [23].

**Fig. 2:**
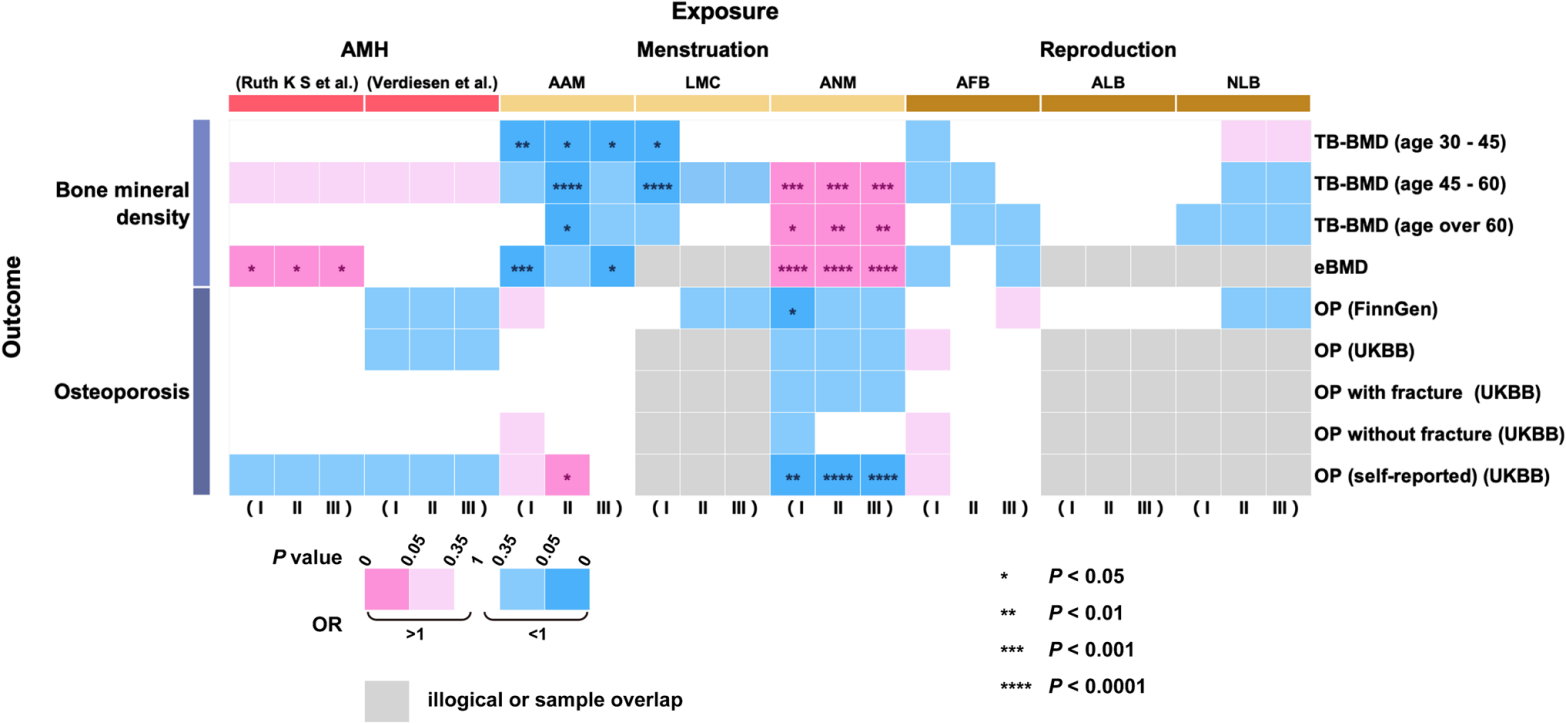
Univariate Mendelian randomization association of genetic liability to AMH and women’s AMH levels and reproductive factors with OP. *OR* odds ratio; (I) Represents confounders not eliminated; (II) Represents SNPs associated with physical status such as “height”, “BMI”, “body fat percentage”, and “rheumatoid arthritis” were excluded; (III) Represents SNPs related to lifestyle and social factors such as “smoking”, “alcohol consumption”, “sleep”, “exercise”, and “education” were excluded

It is worth noting that in the event of sample overlap, we do not include these in our calculations; instead, we utilize additional supplementary data for the computation. Those calculations affected by sample overlap are indicated by the gray squares in Fig. 2**.**

The estimation of eBMD is based on the Quantitative Ultrasound Index through the calcaneus, and the total body bone mineral density (TB-BMD) is determined using dual-energy X-ray absorptiometry (DXA) [23]. Data on OP were coded to the ICD-10 International Statistical Classification of Diseases and Related Health Problems, with OP (FinnGen) and OP (UKBB) coded as M13, OP without fracture (UKBB) coded as M80, and OP without fracture (UKBB) coded as M81. Although the OP (self-reported) (UKBB) data were gathered from the patient’s descriptions, they were verified by nurses and doctors. It is worth mentioning that all outcome factors were analyzed independently with no interference between them. Details of the relevant data are listed in Table 1.

### Statistical Analysis

The random-effects inverse-variance weighted (IVW) method was used as the main statistical analysis method in this study, supplemented by MR-Egger and weighted median analyses as the methods of validation. Only the results of the IVW analysis are shown; the results of the other 2 methods are available in the original data. Heterogeneity was assessed using Cochran’s Q test (“mr_heterogeneity” function), and outliers were identified by the “ivw_radia” function and were removed. When only one SNP was stored, the Wald ratio method was used for analysis. Gene-level pleiotropy was tested by the “mr_pleiotropy” function with the MR-Egger intercept to assess whether the IVs affected outcomes through pathways other than the exposures. The directionality of the results was verified using the MR-Steiger test. Leave-one-out (LOO) sensitivity analysis was performed to determine whether any of the SNPs resulted in misleading or exaggerated effects. All analyses were performed using the TwoSampleMR package (version 0.5.7) in R. MVMR analyses were also conducted to determine the interactions between multiple exposures and OP and BMD (R version 4.2.1).

## Results

### UVMR: Association of Genetic Liability to Women’s AMH Levels and Reproductive Factors with OP

UVMR analyses were first performed for each exposure and outcome to identify confounding factors affecting the outcomes, and then SNPs that were associated with these confounders were excluded in batches. In the first run, SNPs associated with physical status such as “height”, “BMI”, “body fat percentage”, and “rheumatoid arthritis” were excluded [24–26]. SNPs related to lifestyle and social factors such as “smoking”, “alcohol consumption”, “sleep”, “exercise”, “television watching” and “education” were excluded in the second run [27–30]. The results of the three MR analyses (determined using the IVW random-effects model) are summarized in Fig. 2**.** It is worth mentioning that when there were sample overlaps between the exposure factors and outcomes, we did not perform further calculations, which are represented by the gray square in Fig. 2.

**Fig. 3:**
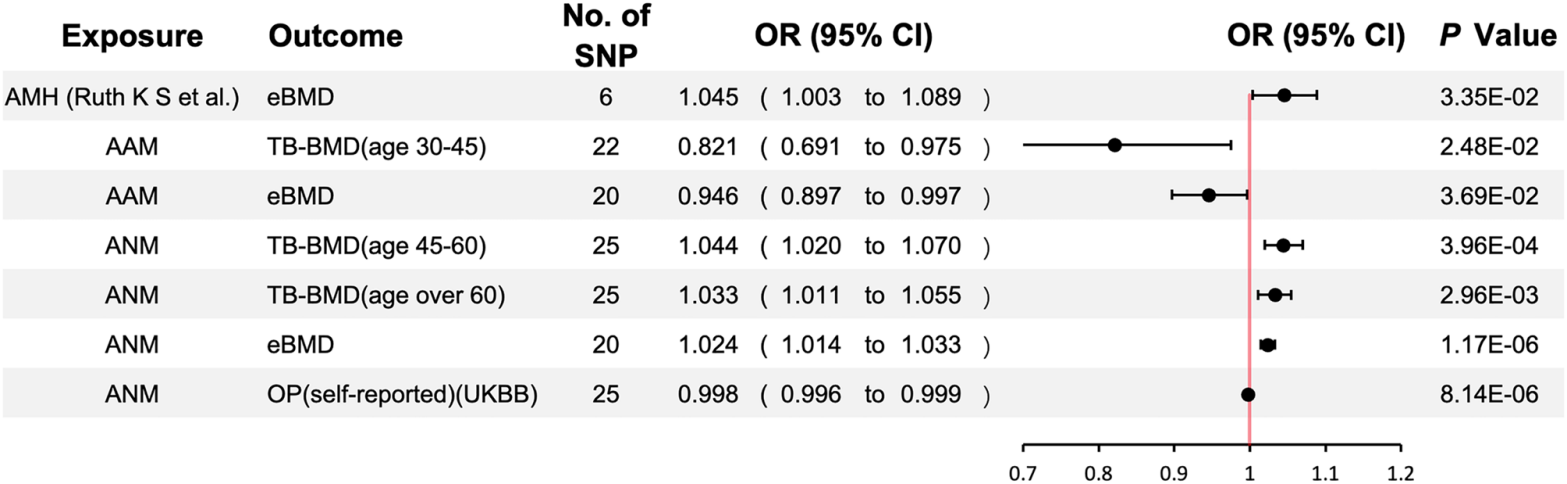
Univariate Mendelian randomization association of genetic liability to AMH, AAM, and ANM with OP. *OR* odds ratio; *CI* confidence interval. Significant at the Bonferroni-corrected threshold of *p*-value < 0.05

After the removal of confounders, our data showed that AMH levels were overall positively correlated with BMD and negatively correlated with OP. Only AMH level (Ruth K S et al.) was causally associated with eBMD [*p*-value < 0.05, OR 95% CI 1.045 (1.003–1.089)]. A similar trend was also observed for ANM. After controlling for covariates, ANM was shown to be significantly and positively linked to TB-BMD (age 45–60) [*p*-value < 0.001, OR 95% CI 1.044 (1.020–1.089)], TB-BMD (age over 60) [*p*-value < 0.01, OR 95% CI 1.033 (1.011–1.055)], and eBMD [*p*-value < 0.0001, OR 95% CI 1.024 (1.014–1.033)] but negatively associated with OP (self-reported) (UKBB) [*p*-value < 0.0001, OR 95% CI 0.998 (0.996–0.999)]. In general, AAM and LMC were negatively associated with BMD and positively correlated with OP. However, after the removal of confounders, only AAM demonstrated a negative causal association with TB-BMD (age 30–45) [*p*-value < 0.05, OR 95% CI 0.821 (0.691–0.975)] and eBMD [*p*-value < 0.05, OR 95% CI 0.946 (0.897–0.997)], and no significant association with the onset of OP. In contrast, there was no significant correlation between LMC and BMD. Furthermore, we did not identify any significant association of AFB, ALB, and NLB with BMD and OP. All positive results after removing confounding factors are shown in Fig. 3**.**

### UVMR: Association of Genetic Liability to AMH with OP After Correcting for Interferences Between Exposures

**Fig. 4:**
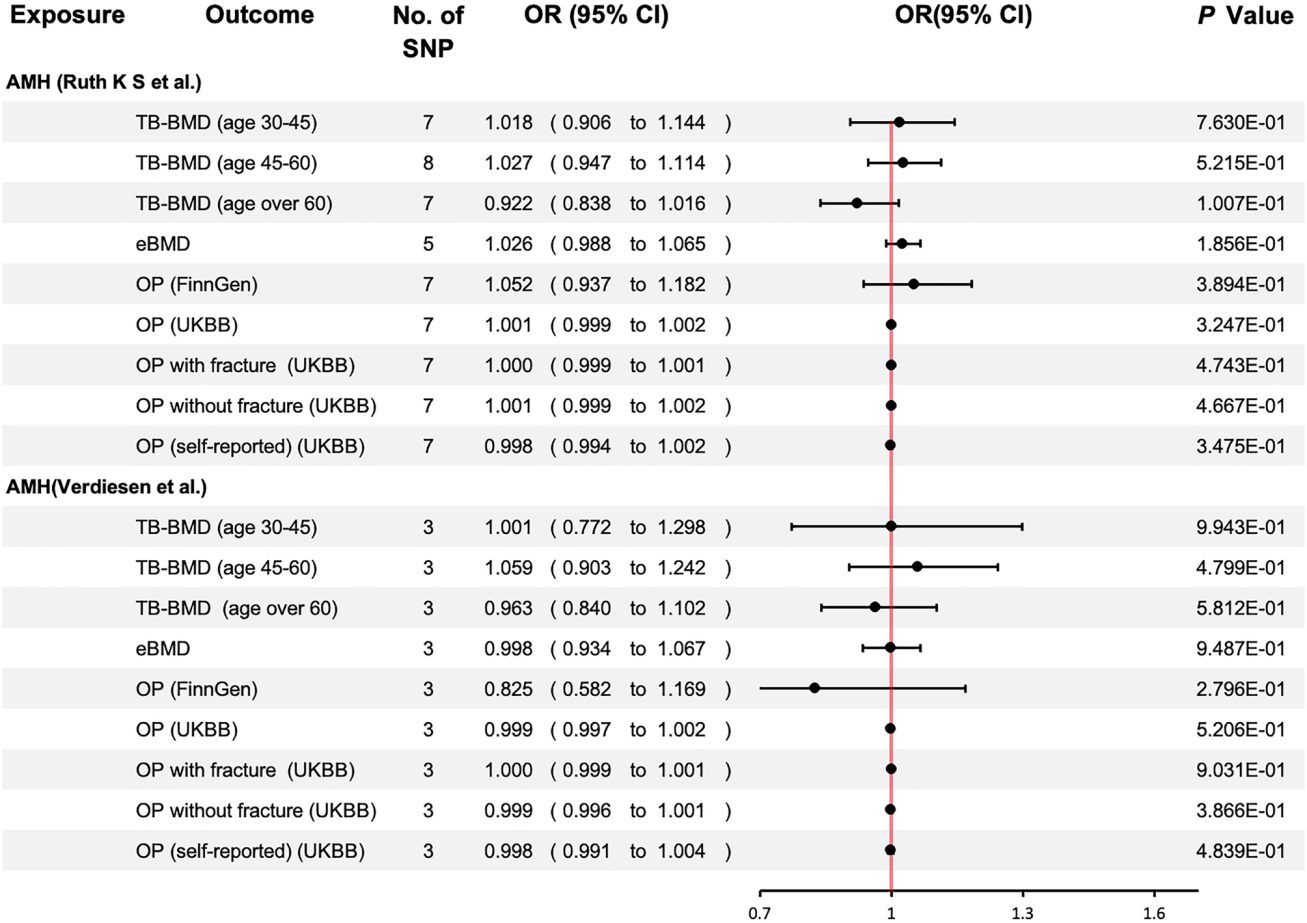
Univariate Mendelian randomization association of genetic liability to AMH with OP after adjusting for the influence of ANM. *OR* odds ratio; *CI* confidence interval. Significant at the Bonferroni-corrected threshold of *p*-value < 0.05

The above results suggest that AMH, AAM, and ANM may be causally associated with BMD or OP. When we further examined the relationship between the exposures, we found that rs16991615 was included in both IVs of AMH, and was highly correlated with ANM (*p*-value = 1.60e−89) [19]. None of the remaining SNPs were found to be correlated with other exposures at the time of this analysis (June 30, 2023). After the removal of rs16991615, our analyses revealed no significant genetic causation between AMH and BMD or OP, which suggested that the initial finding of causality was influenced by ANM (Fig. 4).

### MVMR: Association of Genetic Liability to AAM and ANM with OP

Our UVMR analysis hinted that AAM and ANM may have a significant causal relationship with BMD and OP, but we subsequently found that many SNPs were shared among the IVs of AAM, LMC, and ANM. We also discovered that these SNPs were highly correlated with all of these parameters, which may have introduced bias in the results. Since the confounding effects of these SNPs could not be simply corrected by exclusion, we subsequently performed an MVMR analysis to delineate the direct effect of each of these three exposures on OP. Because the IVs for LMC originated from the same database as those for the outcomes, the analysis was carried out in 2 consecutive runs to avoid sample overlap interference.

**Fig. 5:**
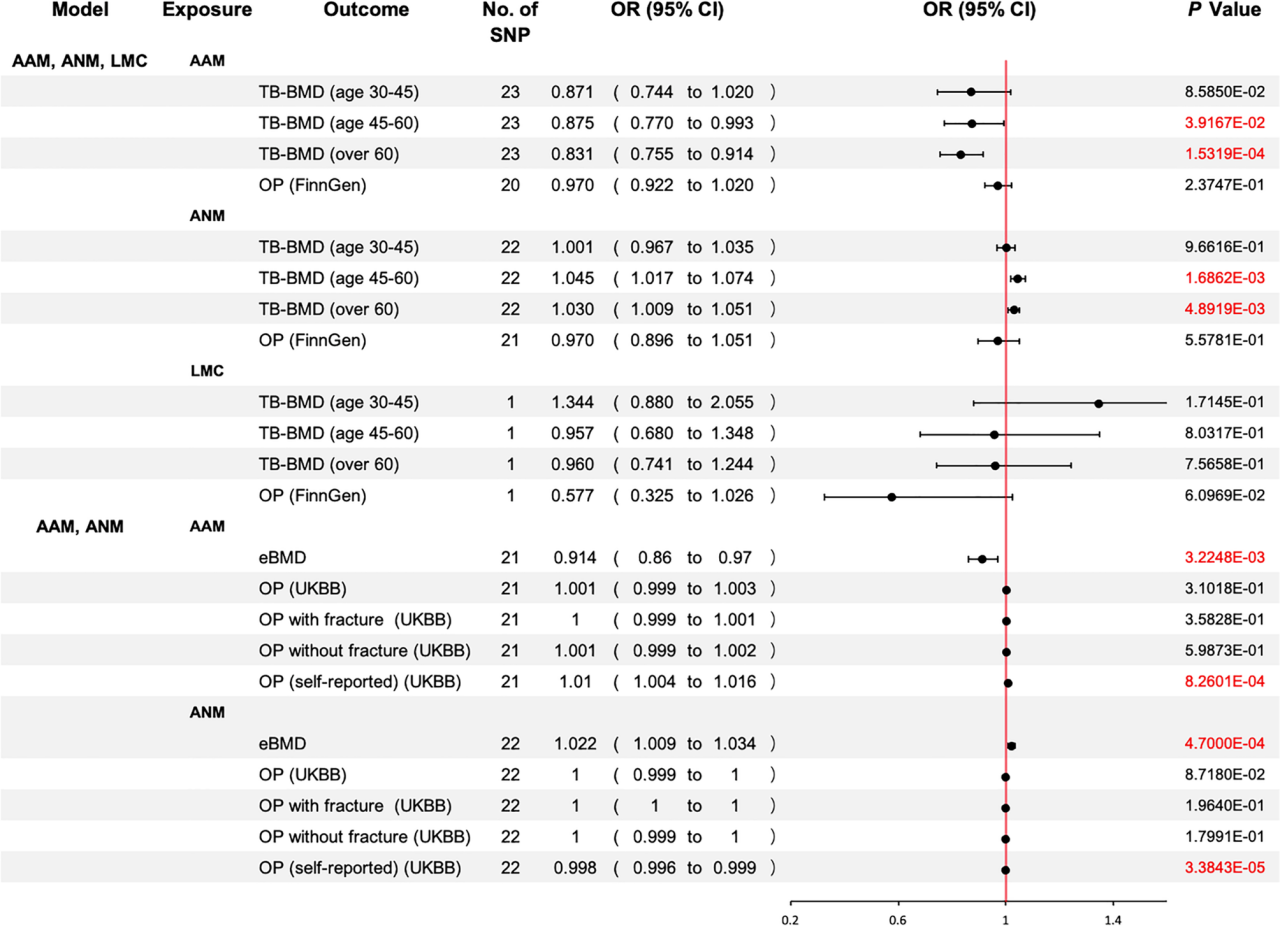
Multivariate Mendelian randomization association of genetic liability to AAM, LMC, and ANM with OP. *OR* odds ratio; *CI* confidence interval. Significant at the Bonferroni-corrected threshold of *p*-value < 0.05

Combined results from the 2-steps analyses revealed that even after adjusting for the effects of AAM and ANM, there was still no significant genetic causation between LMC and BMD or OP. In contrast, after correcting for LMC and the reciprocal effects of AAM and ANM, AAM and ANM were still associated with significant genetic susceptibility to BMD and OP. AAM was negatively associated with TB-BMD (age over 45) and eBMD and positively associated with OP, while the opposite was true for ANM (Fig. 5). It is worth noting that the significant associations of AAM and ANM with OP were based on the self-reported OP cases, and such association was absent when the outcomes were OP (with or without pathologic fracture) diagnosed according to stringent diagnostic criteria.

## Discussion

We performed a comprehensive and detailed MR analysis of AMH levels and female reproductive factors in OP risk. Because exposures to specific genetic variants are randomly assigned at conception, MR studies are analogous to randomized controlled trials (RCTs). Although two-sample MR is an effective method for assessing causal inferences between exposures and outcomes based on pooled data, there are certain limitations. It should also be noted that findings from MR studies should be interpreted with caution, as they can only provide evidence of causal effects when the three key assumptions of IVs are met [15]. When researchers in the past tried to fulfil the exclusion restriction assumption, they were constrained by the state of research at the time and literature availability, which can lead to some bias in the results due to the pleiotropic nature of the SNPs. As evidenced in the current study, the exclusion of one single SNP from the IVs of AMH was sufficient to alter the study results. Therefore, we subsequently conducted MVMR analyses to adjust for the pleiotropic effects of multiple genetic variants by including the phenotype as an additional exposure [13].

Previous observational studies have revealed a significant relationship among AAM, ANM, years of menstruation, and the prevalence of OP [31]. With the substantial secretion of estrogen during puberty, a woman experiences her first menstrual period. It has been reported that female BMD, particularly lumbar spine BMD, increases significantly before and after menarche. On the other hand, ovarian function and estrogen secretion decrease around the time of menopause, resulting in a loss of bone mass [32]. Consistent with this finding, we showed that later AAM and earlier ANM were genetically associated with reduced BMD in women over the age of 45. In addition, despite no evidence of a significant causality in our study, we observed that a longer LMC (implying fewer menstrual periods within the same reproductive lifespan) was associated with a decrease in bone loss in women over 45 years of age, which was in line with previous findings [33]. Collectively, these results demonstrate that the cumulative number or duration of menstruation plays a vital role in female bone metabolism.

It is worth noting that our findings only suggest a significant association of menstrual cycle with decreased BMD and self-reported OP but not with definitively diagnosed OP. The World Health Organization (WHO) has established diagnostic criteria for OP based on the BMD T score, which defines OP as a T score of 2.5 standard deviations (SD) below the mean in young adult women and osteopenia (low bone mass) as 1.0 SD below the mean [32]. Therefore, we speculate that the effect of the menstrual cycle on female BMD may not be adequate for achieving clinically confirmed OP, and the self-reported OP data may be biased by the patients' lack of understanding of the OP diagnostic criteria. Unfortunately, we could not obtain the specific BMD values of the included patients for further investigation.

The effect of menstruation on BMD stems from the fact that both ovulation and bone remodeling in women are related to periodic hormonal changes in the body [34]. During the follicular phase, ovarian granulosa cells produce estrogen, which promotes endometrial development and regulates bone metabolism. Estrogen deficiency increases the expression of receptor activator of nuclear factor kappa B ligand (RANKL), leading to increased osteoclast activation and bone resorption [5, 32]. During the luteal phase, progesterone released by the corpus luteum can also synergize with estrogen to induce osteoblast proliferation [35]. Later AAM and earlier ANM are indicators for lower estrogen or progesterone levels, and some scholars opined that lifetime cumulative exposure to estrogen can prevent OP [31]. In addition, follicle-stimulating hormone (FSH) is the primary factor that stimulates estrogen production by granulosa cells and is regulated by the level of estrogen via negative feedback. FSH has been reported to have a direct and estrogen-independent regulatory effect on osteoclasts and high FSH levels are associated with declined ovarian functions [36].

AMH is a key hormone closely related to the female menstrual cycle and reproductive behaviors. Compared with estrogen, FSH, and other ovarian parameters, AMH is the best predictor for early ovarian decline [37]. The blood levels of AMH peak during the reproductive years of women and then decline gradually to undetectable levels after menopause [38], which is very similar to the change in BMD with age in women [39]. Therefore, AMH may also potentially affect female bone metabolism. One observational study showed that AMH levels can identify women at high risk of bone loss, with each 50% decline in AMH levels resulting in an additional 0.22% loss of spinal BMD in premenopausal women, 0.43% loss in early perimenopause women, and 0.5% loss in late perimenopause women. The authors concluded that AMH could serve as an indicator for women who are on the verge of severe bone loss and guide early intervention. However, this study did not normalize confounding factors such as estrogen levels, AAM, and ANM and failed to show the direct impact of AMH on bone loss [7]. Another prospective study indicated that high FSH and low AMH levels were associated with lower BMD, implying that impaired ovarian function was associated with lower BMD. Though, the findings were only applicable to endometriosis patients [40].

In the present study, we conducted several MR analyses to eliminate factors that could lead to bias. Our preliminary UVMR study showed that AMH levels are negatively correlated with OP and positively associated with eBMD levels. However, after correcting for the effect of ANM, this correlation was no longer present. Given that AMH has been suggested as a clinical predictor for ANM [41] and ANM was significantly linked to BMD decline in women, we hypothesized that the initial associations we found between AMH and OP, as well as eBMD, are very likely due to the influence of AMH on ANM and the results of the previous observational study might have also been confounded by the effect of ANM. AMH has been shown to inhibit the expression of FSH-dependent aromatases and regulate the ovarian response to FSH, thereby inhibiting primary follicle growth. Furthermore, AMH prevents the activation of key genes for steroidogenesis (CYP19A1 and P450scc) and was found to be negatively correlated with estrogen levels in human follicular fluid [42, 43]. When ovarian reserve function is reduced, FSH directly suppresses AMH or inhibits oocyte growth factor-mediated AMH synthesis [44]. It is evident that there is an association among estrogen, FSH, and blood AMH levels, but the mechanisms by which these factors interact remain unclear. However, we must point out that the fewer GWAS and limited number of IVs available for AMH in our study may potentially confound our MR result. Hence, more genetic variants for AMH will need to be identified to verify our findings.

Due to hormones associated with the menstrual cycle, female BMD significantly increases after the onset of menstruation and subsequently fluctuates with age. Although the exact age at which female BMD peaks is debated, most researchers believe that female TB-BMD peaks between the ages of 25 and 35 years and then decreases annually thereafter [39]. In addition to aging, childbearing may also impact female bone metabolism. The only source of calcium for growing fetal bones during pregnancy is maternal bone minerals, which poses a challenge for female BMD. In response to fetus-induced bone mass loss, the maternal body undergoes adaptive changes by doubling the calcium absorption in the intestine [45]. Therefore, it is worthwhile to explore the effect of childbearing on changes in BMD and the risk of OP in women. Previous studies on this topic have mainly focused on parity. A cross-sectional study of Korean women showed that a higher number of births can increase the risk of OP-related fractures in women, and the same results were also reported in observational studies of women in rural China and India [8–10]. Although these studies adjusted for multiple confounding factors, they failed to control for the effects of AAM and ANM on BMD. Consequently, the effect of parity on BMD may be ascribed to its effect on the menstrual cycle. Interestingly, our MR analysis of AFB, ALB, and NLB revealed that these fertility-related factors were not directly linked to BMD and OP, suggesting that there may have been bias in the previous findings. Nonetheless, a limitation in our analysis is the inability to obtain the specific age of the population from which the ALB and NLB data were derived, and hence further comprehensive and in-depth studies are warranted.

Our study has several significant advantages. First, this is the first study to use an MR approach to explore the causal relationship between AMH levels and OP in women. Second, we included six reproductive factors as exposures, which rendered our analysis more comprehensive than that of previous studies. Finally, we included data from multiple large-scale GWAS and utilized two-sample MVMR to ensure the reliability of the causal relationship between the exposure factors and outcomes. At the same time, we acknowledge that our study has some potential biases. For example, although we endeavored to restrict our study population to Europeans, the genetic diversity among populations across different European countries may still lead to bias caused by population stratification. Unfortunately, existing data do not support our ability to further focus our study on specific countries or races. Second, some data were missing from the included cohorts. The age range of the populations for LMC, ALB, and NLB data were not available to us. Although this limitation does not affect the computational aspects of our study, it potentially undermines the scientific validity of the inferences based on these metrics. It is important to emphasize that for the majority of data included in our study, the age range of population samples is specified. Furthermore, while the focus of our study was on females and most of the data included were from female participants, due to issues such as sample overlap, we had to incorporate a small amount of mixed-sex data for complementary analysis. Lastly, despite our efforts to comprehensively consider all potential confounding factors, there may still be unaccounted confounders that could introduce bias into our findings. It should be noted that the potential biases mentioned are not a result of our subjective decision-making but primarily of the GWAS data limitations currently available. We will continue to monitor developments in related research to update our findings.

## Conclusion

Our study suggests that AMH levels, length of menstrual cycle (LMC), age at first birth (AFB), age at last birth (ALB), and number of live births (NLB) are not significantly associated with BMD changes and OP. Later age at menarche (AAM) and earlier age at natural menopause (ANM) are significantly causally associated with decreased BMD, but not with OP. This indicates that a reduction in total lifetime exposure to estrogen may negatively impact BMD; however, this influence alone is insufficient to induce OP without the concomitant action of other factors.

Our findings underscore the importance of early detection and intervention in potential bone loss throughout a woman’s life cycle, particularly in those with later menarche and earlier menopause. Strategies such as hormone replacement therapy for premature menopause, physical exercise from puberty to premenopause, and calcium and vitamin D supplementation may mitigate the potential negative impact on bone health. However, the direct effects of these strategies on OP and their interaction with ANM or AAM in influencing bone quality remain to be further elucidated. It is imperative to deepen our understanding of more factors affecting changes in female BMD to enhance women’s bone health and prevent OP more effectively.

## Data Availability

Raw data were extracted from the MRC-IEU database (https://gwas.mrcieu.ac.uk/), the UK Biobank database (https://www.nealelab.is/uk-biobank), the FinnGen database (https://www.finngen.fi/fi), and other original studies (Details are in Table 1).

## Abbreviations

OP: Osteoporosis
BMD: Bone mineral density
eBMD: Heel bone mineral density
TB-BMD: Total body bone mineral density
MR: Mendelian randomization
SNPs: Single nucleotide polymorphisms
IVs: Instrumental variables
UVMR: Univariate Mendelian randomization
MVMR: Multivariate Mendelian randomization
IVW: Inverse-variance weighted
OR: Odds ratio
CI: Confidence interval
AMH: Anti-Müllerian hormone
AAM: Age at menarche
ANM: Age at natural menopause
LMC: Length of menstrual cycle (the interval between two periods)
AFB: Age at first birth
ALB: Age at last birth
NLB: Number of live births
FSH: Follicle-stimulating hormone
